# Low tissue levels of miR-125b predict malignancy in solitary fibrous tumors of the pleura

**DOI:** 10.1186/s12931-017-0528-7

**Published:** 2017-03-02

**Authors:** Matthias Brock, Selma Hottinger, Matthias Diebold, Alex Soltermann, Wolfram Jochum, Malcolm Kohler, Lars C. Huber, Daniel P. Franzen

**Affiliations:** 1 Department of Pulmonology, University Hospital Zurich, University of Zurich, Rämistr. 100, 8091 Zurich, Switzerland; 2 Department of Pathology and Molecular Pathology, University Hospital Zurich, University of Zurich, Zurich, Switzerland; 3 Institute of Pathology, Cantonal Hospital St Gallen, St Gallen, Switzerland

**Keywords:** microRNA, Solitary fibrous tumor, Pleura, Biomarker

## Abstract

**Background:**

Solitary fibrous tumors of the pleura (SFTP) are rare neoplasia of the chest. A subset of SFTP follows a malignant course, sometimes several years after complete resection. Traditional scoring systems based on clinical and histological features are poor predictors of biological behavior. This study aimed to investigate tumor-associated miRNAs expression as novel biomarkers to predict the clinical behavior of SFTP.

**Methods:**

Formalin-fixed and paraffin-embedded SFTP tissues blocks from patients surgically resected between 1992 and 2013 at two tertiary care teaching hospitals were included. SFTP tumors were categorized as either malignant or benign variants according to the WHO classification. Following miRNAs levels were measured: let-7a, miR-16b, miR-17, miR-21, miR-31, miR-34a, miR-92a, miR-125a, miR-125b, miR-195-5b, miR-203a, and miR-223. Differential gene expressions which were calculated with the threshold cycle (C_t_) method were compared among the two variants.

**Results:**

Thirty-eight patients (40% male, mean age 62.2 (±10.9) years) were included. Expression levels of miR-125b showed a significant difference between benign compared to malignant variants (−3.08 ± 0.93 vs. -2.22 ± 1.36, *p* = 0.0068). Furthermore, lower levels of miR-125b were found to be associated with increased tumor size (*p* = 0.0414). Thus, downregulation of miR-125b indicates malignant transformation. All other investigated miRNAs were not associated with grading of SFTP.

**Conclusions:**

Our data suggest a potential role of miR-125b in the pathogenesis of tumor growth and malignant transformation of SFTP, respectively. Further studies have to address the potential use of miRNA-125b as a biomarker or therapeutic target in SFTP.

## Background

Solitary fibrous tumors of the pleura (SFTP) are rare neoplasia within the chest cavity with an estimated incidence of approximately 0.2/100’000 persons per year [1], and account for 5% of all pleural tumors [2]. Beside the preferred occurrence in the pleura diverse anatomical locations of solitary fibrous tumors have been reported [3, 4]. Historically, SFTP were denominated as “localized mesothelioma” reflecting the lack of consensus with respect to pathogenesis and biological behavior of these tumors [5]. Nowadays, SFTP have been shown to arise from mesenchymal cells and, as such, express immuno-histochemical markers including vimentin and CD34 [6, 7]. The majority of SFTP follow a benign course with a high cure rate after complete surgical resection [8, 9]. However, malignant variants with a high risk of local recurrence, metastases, and increased mortality are found in 7–60% [9–11]. In addition, some SFTP which were initially assessed to follow a benign course may transform into malignant variants, sometimes several years after R0-resection [12, 13]. Therefore, characteristics that distinguish benign from malignant variants are crucial for estimating the risk of an adverse outcome, and planning adjuvant therapies and adequate follow-up examinations [5].

England et al. were the first to describe six clinico-pathologic features predicting a malignant behavior. These risk factors included tumor size, localization, sessile tumor, existence of necrosis or hemorrhage, high mitoses count, and evidence of nuclear pleomorphism [9]. More recently, a staging system based on tumor seating and on the above mentioned features have been proposed in order to improve identification of malignant variants [14]. However, several multivariable models identified these characteristics which are based exclusively on clinico-pathologic features as poor predictors for the biological behavior of SFTP [15–17]. Yet, there is insufficient data on immune-histochemical and molecular markers predicting the outcome of SFTP.

MiRNAs are small non-coding RNA fragments of 20–22 nucleotides in length that are commonly associated with gene silencing. Since there is growing evidence that miRNAs might be of major interest in the diagnosis and prognosis of different diseases including lung cancer [18] and mesothelioma [19], this study aimed to investigate the role of miRNAs as biomarkers to predict the clinical behavior of SFTP.

## Methods

### Patients and sample collection

Consecutive patients undergoing surgical resection of SFTP between 1992 and 2013 at two tertiary care teaching hospitals and with a follow up of at least one month were eligible for the study. The diagnosis of SFTP was made by the Departments of Pathology of either of these institutions, based on the typical morphologic and immuno-histochemical features [9]. Patient data on demographics, clinical presentation, radiologic features and surgical treatment as well as follow-up data were extracted from medical records and, if required, obtained from the patients’ general practitioners. Formalin-fixed and paraffin-embedded (FFPE) tissue blocks of all tumors were collected, and a representative slide was cut and reviewed by a dedicated pulmonary pathologist (A.S.). Patients with wrong diagnosis of SFTP or missing FFPE tissue block were excluded from the study. According to the WHO classification [20], the tumor samples were allocated to either malignant or benign variants. Thus, the presence of more than one feature of the traditional classification system proposed by England et al. [9], including tumor size greater 10 cm, atypical localization, sessile morphology, existence of necrosis or hemorrhage, more than four mitoses per 10 HPF and nuclear polymorphism were used to define malignant SFTP.

### MiRNA Isolation

Total RNA including miRNAs was purified from FFPE lung tissue slides using the miRCURY RNA Isolation Kit (Exiqon, Vedbaek, Denmark) as recommended by the manufacturer. Briefly, human lung tissue was scraped off from FFPE tissue slides (four slides of 10 μm from each patient) with a scalpel and placed in Eppendorf tubes. To remove paraffin from the sections, paraffin dissolver was added. Next, samples were centrifuged and the supernatant was removed without disturbing the pellet. Then Proteinase K was added followed by an incubation step for 30 min at 56 °C. After addition of precipitation buffer, the samples were incubated for five minutes on ice. Next, samples were centrifuged before the supernatant was transferred to a new tube and incubated for another 15 min at 80 °C. To adjust binding conditions, binding buffer was added and incubated for 1 min. Each sample was loaded on a column to bind the RNA to the column. The samples were passed through a filter cartridge, centrifuged and the flow through was discarded. After addition of wash buffer to each column, the samples were again centrifuged. As a following step, DNA was digested by the addition of DNase I mix to the center of each filter and incubated for 15 min before washed twice. This step was necessary to remove DNA that would otherwise interfere with miRNA quantification. Next, RNase-free water was applied to the center of each filter and the RNA containing samples were centrifuged to pass the mixture through the filter. The eluted RNA was then analyzed for miRNA expression or stored at −80 °C.

### Selection and quantification of mature miRNAs

The first step of this study was focused on the identification of dysregulated genes in SFTP. MiRNAs that potentially interact with these genes were retrieved using PubMed search (Table 1). As such, the levels of the following miRNAs were measured: let-7a, miR-16b, miR-17, miR-21, miR-31, miR-34a, miR-92a, miR-125a, miR-125b, miR-195-5b, miR-203a, and miR-223. Mature miRNAs were detected by specific stem-loop primers and reverse transcribed using GoScript reverse transcriptase (Promega, Dübendorf, Switzerland). Quantification of complementary DNA (cDNA) was performed using SYBR Green quantitative PCR (qPCR, Applied Biosystem, StepOnePlus system, Life Technologies, Zug, Switzerland). Sequences of primers used in this study for reverse transcription and amplification of miRNAs are shown in Table 2. Specific amplification of the miRNA of interest was confirmed by melt curve analysis. Differential gene expression was calculated with the threshold cycle (C_t_) method [21]. MiRNA expression levels are shown in Δc_t_ values normalized to the levels of RNU48 and RNU49. Higher Δc_t_ values indicate lower abundance of the miRNA of interest and vice versa.

**Table 1:** Protein targets and corresponding miRNAs

Target	miRNA	Verification by qPCR	Reference
MIB-1 (Ki-67)	miR-21	yes	Rask et al. [36]
	miR-519d	yes	Hou et al. [37]
p16 or p53	hsa-miR-31	yes	Robinson et al.[38, 39]
	let-7a	no	Akaike et al. [40]
	hsa-miR-125b	yes	Liu et al. [41]
	hsa-miR-16	yes	Feng et al. [42]
	hsa-miR-17	n/a	Toledo et. [43]
	hsa-miR-195	yes	Flavin et al. [44]
	hsa-miR-203	n/a	Guled et al. [45]
	hsa-miR-92a	yes	Dews et al. [46]
	hsa-miR-34a	yes	Ji et al. [47]
STAT6	hsa-miR-361-5p	yes	Chung et al. [48]

*qPCR* quantitative real-time polymerase chain reaction, n/a not available

**Table 2:** Primer sequences

Primer	miRNA	Sequence
Reverse	Universal rev	5’ - GAG GTA TTC GCA CTG GAT AC - 3’
RT	RNU-48	5’ - GTC GTA TCC AGT GCA GGG TCC GAG GTA TTC GCA CTG GAT ACG ACG GTC AG - 3’
Forward	RNU-48	5’ - CCA TGA GTG TGT CGC TGA TG - 3’
RT	RNU-49	5’ - GTC GTA TCC AGT GCA GGG TCC GAG GTA TTC GCA CTG GAT ACG ACA ATC AG - 3’
Forward	RNU-49	5’ - AAG CGA TAA CTG ACG AAG ACT AC - 3’
RT	let-7a	5’ - GTC GTA TCC AGT GCA GGG TCC GAG GTA TTC GCA CTG GAT ACG ACA ACT AT - 3’
Forward	let-7a	5’- CGG TGA GGT AGT AGG TTG TAT - 3’
RT	miR-16b	5’ - GTC GTA TCC AGT GCA GGG TCC GAG GTA TTC GCA CTG GAT ACG ACC GCC AA - 3’
Forward	miR-16b	5’- CCT AGC AGC ACG TAA ATA TTG G - 3’
RT	miR-17	5’ - GTC GTA TCC AGT GCA GGG TCC GAG GTA TTC GCA CTG GAT ACG ACC TAC CT - 3’
Forward	miR-17	5’ - GCG GCA AAG TGC TTA CAG TG - 3’
RT	miR-21	5’ - GTC GTA TCC AGT GCA GGG TCC GAG GTA TTC GCA CTG GAT ACG ACT CAA CA - 3’
Forward	miR-21	5’ - GCC CGC TAG CTT ATC AGA CTG ATG - 3’
RT	miR-31	5’ - GTC GTA TCC AGT GCA GGG TCC GAG GTA TTC GCA CTG GAT ACG ACA GCT AT - 3’
Forward	miR-31	5’- CAG GCA AGA TGC TGG CAT AG - 3’
RT	miR-34a	5’ - GTC GTA TCC AGT GCA GGG TCC GAG GTA TTC GCA CTG GAT ACG ACA CAA CC - 3’
Forward	miR-34a	5’ - TGG CAG TGT CTT AGC TGG TTG – 3’
RT	miR-92a	5’ - GTC GTA TCC AGT GCA GGG TCC GAG GTA TTC GCA CTG GAT ACG ACA CAG GC - 3’
Forward	miR-92a	5’ - CCC TAT TGC ACT TGT CCC G - 3’
RT	miR-125a	5’ - GTC GTA TCC AGT GCA GGG TCC GAG GTA TTC GCA CTG GAT ACG ACT CAC AG - 3’
Forward	miR-125a	5’ - TCC CTG AGA CCC TTT AAC C - 3’
RT	miR-125b	5’ - GTC GTA TCC AGT GCA GGG TCC GAG GTA TTC GCA CTG GAT ACG ACT CAC AA - 3’
Forward	miR-125b	5’- CTC CCT GAG ACC CTA ACT TG - 3’
RT	miR-195-5b	5’ - GTC GTA TCC AGT GCA GGG TCC GAG GTA TTC GCA CTG GAT ACG ACG CCA AT - 3’
Forward	miR-195-5b	5’- GGT AGC AGC ACA GAA ATA TTG G - 3’
RT	miR-203a	5’ - GTC GTA TCC AGT GCA GGG TCC GAG GTA TTC GCA CTG GAT ACG ACC TAG TG - 3’
Forward	miR-203a	5’- CCG TGA AAT GTT TAG GAC CAC - 3’
RT	miR-223	5’ - GTC GTA TCC AGT GCA GGG TCC GAG GTA TTC GCA CTG GAT ACG ACT GGG GT - 3’
Forward	miR-223	5’- CCC TGT CAG TTT GTC AAA TAC C- 3’

### Statistics

All data are presented as mean ± standard deviation (SD). Parametric or non-parametric distribution of data was determined using the Kolmogorov-Smirnov test. Data comparison between malignant and benign SFTP variants were performed using independent, two-tailed Student’s *t*-test for parametric distribution as well as Mann–Whitney *U* test for nonparametric distribution. Nominal data were compared using the Fisher’s exact test. Correlation analysis was carried out with Pearson’s calculations. Values with a *p* < 0.05 were considered to be statistically significant (*p < 0.05, **p < 0.01, ***p < 0.001). All statistical calculations were performed using the software package GraphPad Prism Version 5.0 (GraphPad Software, San Diego, CA, USA).

## Results

**Fig. 1:**
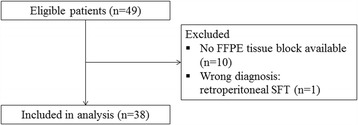
Study flowchart. FFPE, formalin-fixed and paraffin-embedded; SFT, solitary fibrous tumor

In total, 38 patients (40% male, mean age 62.2 (±10.9) years) were included (Fig. 1). Baseline characteristics as well as the traditional pathological features of SFTP published by England et al. [9] are summarized in Table 3. Sixteen patients were found to have malignant variants according to completion of two, three, or four to five of these features in seven, four and five patients, respectively.

**Table 3:** Patient characteristics and features of solitary fibrous tumors of the pleura, *n* = 38

Characteristics	All, *n* = 38	Malignant, *n* = 16	Benign, *n* = 22	*p*-value*
Male sex	15 (40)	9 (56)	6 (27)	0.09
Age, years	62.2 (10.9)	64.7 (9.2)	60.3 (11.8)	0.24
Sessile morphology	15 (42)	11 (69)	4 (20)	0.006
Nuclear pleomorphism	6 (16)	4 (25)	2 (9)	0.22
Necrosis or hemorrhage	11 (29)	8 (50)	3 (14)	0.028
Tumor size, cm	10.6 (6.7)	13.6 (7.3)	8.3 (5.3)	0.014
Mitoses, n per HPF	4.1 (5.4)	45 (37)	1 (1)	<0.0001
>4 mitoses per HPF	11 (28.9)	11 (68.8)	0 (0)	<0.0001

Data presented as mean (± SD) or n (%) as appropriate

*HPF* high-power field

*Statistical analysis by unpaired student’s *t*-test or Fisher’s exact test as appropriate

### Detection and quantification of miRNAs in SFTP

**Fig. 2:**
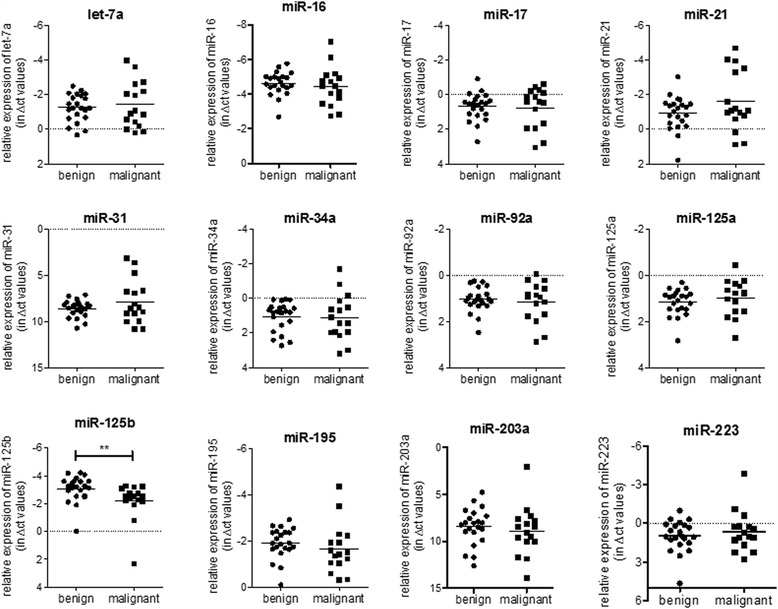
The expression profile of selected miRNAs in FFPE lung tissue slides from SFTP samples. The relative expression levels of selected miRNAs (let-7a, miR-16b, miR-17, miR-21, miR-31, miR-34a, miR-92a, miR-125a, miR-125b, miR-195-5b, miR-203a, and miR-223) from lung tissue slides of benign (*n* = 22) and malignant (*n* = 16) SFTP samples is shown. The obtained C_t_ values of miRNAs were normalized to the expression levels of the mean of the endogenous controls RNU48 and RNU49. Statistical analysis by unpaired student’s *t*-test or Mann–Whitney *U* test. **p < 0.001

The mean expression levels of all measured miRNAs are presented in Table 4 and Fig. 2, separated in benign and malignant SFTP variants according to England et al. [9]. Calculation of fold change difference and statistical analysis is provided in Table 4. When assessing the expression levels in benign and malignant variants, substantial changes were found for different miRNAs. However, due to high variability between the samples, only alterations in the expression levels of miR-125b reached statistical significance (miR-125b in benign SFTP: −3.08 ± 0.93, miR-125b in malignant SFTP −2.22 ± 1.36, *p* = 0.0068, Fig. 2). Thus, all further experiments were performed on miR-125b.

**Table 4:** Expression levels of miRNAs in SFTP

miRNA	C_t_ values in benign SFTP	C_t_ values in malignant SFTP	*P* values	Fold change
let-7a	−1.24 ± 0.79	−1.42 ± 1.33	0.61	1.13
miR-16-5p	−4.62 ± 0.67	−4.43 ± 1.14	0.51	0.87
miR-17	0.7 ± 0.78	0.77 ± 1.16	0.67	0.95
miR-21	−0.93 ± 0.97	−1.61 ± 1.75	0.47	1.6
miR-31	8.61 ± 0.91	7.89 ± 2.37	0.2	1.65
miR-34a	1.05 ± 0.83	1.1 ± 1.32	0.69	0.96
miR-92a	1.06 ± 0.53	1.16 ± 0.86	0.65	0.93
miR-125a	1.18 ± 0.56	0.99 ± 0.78	0.38	1.14
miR-125b	−3.08 ± 0.93	−2.22 ± 1.36	0.007	0.69
miR-195-5p	−1.9 ± 0.66	−1.66 ± 1.08	0.39	0.84
miR-203a	8.45 ± 1.94	8.91 ± 2.62	0.54	0.73
miR-223	0.99 ± 1.21	0.63 ± 1.57	0.93	1.29

### Levels of miR-125b

**Fig. 3:**
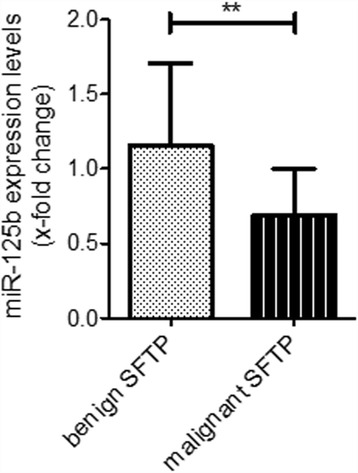
The expression of miR-125b is significantly reduced in malignant SFTP samples. The expression of miR-125b in benign (*n* = 22) and malignant SFTP samples (*n* = 16) was measured by qPCR analysis. Statistical analysis by unpaired student’s *t*-test. ** *p* < 0.001

**Fig. 4:**
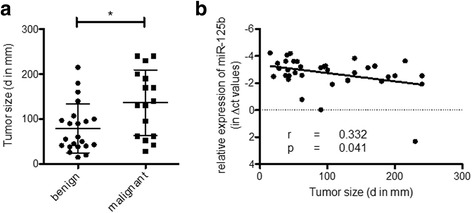
Significant inverse correlation between miR-125b levels and tumor size in SFTP. **a** The size of tumors in benign (*n* = 22) and malign SFTP (*n* = 16) presented as diameter in mm is shown. **b** Correlation analysis of miR-125b levels with the size of tumors in SFTP. Statistical analysis by Mann–Whitney *U* test (**a**). Correlation analysis was carried out with Pearson’s calculations (**b**). *p < 0.05

As shown in Fig. 3, the expression of miR-125b was found to be significantly decreased in malignant SFTP variants (reduction to 69). Next, we analyzed the expression data of miR-125b in the context of the tumor size. In malignant SFTP tumor size was significantly increased when compared to benign samples (diameter of tumors in mm: benign SFTP 79.32 ± 54.32 vs. malignant SFTP 136.3 ± 73.06, *p* = 0.0125 Fig. 4a). When tumor size was compared to miR-125b levels, we found that expression levels of miR-125b inversely correlated with tumor size (Fig. 4b), indicating that lower levels are associated with an increase in tumor size (*p* = 0.0414). These data suggest a potential role of miR-125b in the pathogenesis of tumor growth and malignant transformation of SFTP.

## Discussion

In this translational study, we addressed the pattern and expression levels of miRNAs in SFTP and found that i) reduced levels of miR-125b are indicative of malignant transformation of SFTP and, ii), lower expression of miR-125b was inversely correlated with tumor size.

The challenge of diagnosis and staging of SFTP is the prediction of biological behavior. The majority of SFTP follow a benign course, but a considerable high proportion of these tumors may present with an aggressive behavior including distant metastases or recurrence, sometimes several years after complete resection [9–13, 22, 23]. For this reason, several scoring systems have been introduced which are originally based on six pathological features proposed by England et al. [9, 14]. However, multivariable models identified these features as poor predictors for the biological behavior of SFTP [15–17]. And, clinical parameters do not seem to have sufficient discriminative power to predict the outcome of SFTP [17]. Thus, investigation on immuno-histochemical and molecular markers is needed to characterize SFTP and their outcome more accurately. For example, high p53 expression has been shown to be associated with adverse outcome of SFTP [15, 24]. Recently, we found that Ki-67 labeling index (MIB-1), an immuno-histochemical marker of proliferation, is a promising predictor for an adverse outcome in SFTP [17]. Due to its role in ribosomal RNA transcription [25], the Ki-67 antigen is of special interest in the context of non-coding RNAs profiling. These small RNA fragments have emerged as important gene regulators and it is estimated that more than 50% of the human genome are controlled by non-coding RNAs [26]. Within the group of non-coding RNAs, miRNAs are the best-characterized to date [27]. As such, miRNAs have been implicated in the pathogenesis of disease and as biomarkers for diagnosis and prognosis. Of interest, miRNAs have distinct properties that make them interesting biomarker candidates including stability and standardized quantification within tissues and biofluids [28].

Here we employed a screening approach and assessed the expression levels of selected miRNAs in clinically well-characterized samples of SFTP. Isolation and quantification of miRNAs was performed in formalin-fixed and paraffin-embedded (FFPE) tissue blocks. This method has been described before [29, 30] and was recently confirmed as a feasible technology by our own work using lung tissue slides [31]. These data, together with the results provided here, highlight both the reliability and the reproducibility by which miRNAs are isolated and quantified from FFPE.

Of interest, out of a panel of selected miRNAs that have previously been associated with lung cancer [18], only the expression of miR-125b was found to be significantly altered when benign and malignant variants of SFTP were compared. miR-125b has been identified as an important oncomir [32] that is dysregulated in many different types of cancers including breast cancer [33], hepatocellular and cologastric carcinoma [34] and thyroid neoplasias [35]. Since both up and down regulation of miR-125b have been described in cancer, it was suggested that miR-125b has tissue or cell-type specific effects [32]. In this context, miR-125b was linked to aberrant regulation of several target genes involved in the control of differentiation, proliferation and apoptosis [32]. Whether and which of these mechanisms apply to SFTP is beyond the scope of this descriptive analysis and remains unclear to date. However, since tumor size was significantly correlated with expression levels of miR-125b, our results here provide evidence that, in addition to mediating malignant transformation of SFTP, miR-125b might have pro-proliferative and anti-apoptotic effects on pleural cells.

Our study is mainly limited by its descriptive design and by a relatively low patient number. However, by analyzing a substantial number of samples obtained from two different tertiary care centers, this is the first report on a pathogenetic role of miRNAs in SFTP. Furthermore, since there are concerns regarding the accuracy of the traditional grading systems of SFTP [15–17], miR-125b should be prospectively investigated in a future study with higher sample size. Moreover, the discriminative power of miR-125b should be confirmed with outcome data and other immunohistochemical parameters (e.g. p53 expression or MIB-1 rather than traditional grading systems. However, due to the low sample size we were not able to overcome this limitation.

## Conclusions

Our data show that lower miR-125b expression levels are associated with an increase in tumor size and that downregulation of miR-125b indicates malignant transformation. These data emphasize a role of miR-125b in the clinical course of SFTP. Further studies have to address the potential use of miR-125b as a biomarker or therapeutic agent in SFTP.

## Abbreviations

FFPE: Formalin-fixed and paraffin-embedded
HPF: Highpower field
miRNA: microRNA
SFTP: Solitary fibrous tumor of the pleura
